# Structural Plasticity of the Hippocampus in Neurodegenerative Diseases

**DOI:** 10.3390/ijms23063349

**Published:** 2022-03-20

**Authors:** Poornima D. E. Weerasinghe-Mudiyanselage, Mary Jasmin Ang, Sohi Kang, Joong-Sun Kim, Changjong Moon

**Affiliations:** 1 Department of Veterinary Anatomy and Animal Behavior, College of Veterinary Medicine and BK21 FOUR Program, Chonnam National University, Gwangju 61186, Korea; 208314@jnu.ac.kr (P.D.E.W.-M.); mcang3@up.edu.ph (M.J.A.); shrloveu@gmil.com (S.K.); centraline@chonnam.ac.kr (J.-S.K.); 2 College of Veterinary Medicine, University of the Philippines Los Baños, Los Baños 4031, Philippines

**Keywords:** hippocampal function, neuroplasticity, neurodegenerative diseases, structural plasticity

## Abstract

Neuroplasticity is the capacity of neural networks in the brain to alter through development and rearrangement. It can be classified as structural and functional plasticity. The hippocampus is more susceptible to neuroplasticity as compared to other brain regions. Structural modifications in the hippocampus underpin several neurodegenerative diseases that exhibit cognitive and emotional dysregulation. This article reviews the findings of several preclinical and clinical studies about the role of structural plasticity in the hippocampus in neurodegenerative diseases, including Alzheimer’s disease, Parkinson’s disease, Huntington’s disease, and multiple sclerosis. In this study, literature was surveyed using Google Scholar, PubMed, Web of Science, and Scopus, to review the mechanisms that underlie the alterations in the structural plasticity of the hippocampus in neurodegenerative diseases. This review summarizes the role of structural plasticity in the hippocampus for the etiopathogenesis of neurodegenerative diseases and identifies the current focus and gaps in knowledge about hippocampal dysfunctions. Ultimately, this information will be useful to propel future mechanistic and therapeutic research in neurodegenerative diseases.

## 1. Introduction

Neuroplasticity is the ability of neural networks in the brain to alter through development and rearrangement, and is generally categorized into structural and functional plasticity [1,2,3]. Structural plasticity involves the expansion or retraction of the synaptic area through the remodeling of spines, dendrites, and/or axons [1]. Functional plasticity involves regulation of neurotransmission, reorganization of synaptic components and receptors, and regulation of the strength or efficiency of synaptic transmission [4,5].

Structural plasticity, such as dendritic formation and spine development, is mainly exhibited through the regulation of the neuronal actin cytoskeleton [6,7]. Several actin regulating proteins influence the structural plasticity of neurons by maintaining the equilibrium between G- and F-actin [8]. Furthermore, several signaling cascades are involved in neuronal structural modification, wherein actin and microtubule cytoskeletons serve as the common end targets [9,10]. Guanosine triphosphate hydroxylase (GTPases) is an important family of signaling molecules that transduces extracellular signals to control the actin assembly [11]. Ras-related C3 botulinum toxin substrate 1 (Rac1) and Ras homolog (Rho) family member A (RhoA) are some widely studied members of the Rho family of GTPases. Rac1 promotes spine growth through the activation of p21 (RAC1) activated kinase 1 (PAK1) and LIM domain kinase 1 (LIMK1), while RhoA negatively regulates the spine formation [7]. Two major upstream signals which can trigger GTPases are the brain-derived neurotrophic factors (BDNF) [12,13] and glutamate receptors (GluRs) [14], both of which greatly affect the neuronal micromorphometry [12,14,15]. For example, BDNF promotes the actin polymerization in dendritic spines of hippocampal neurons through Rac1 [13]. The stimulation of group I metabotropic GluRs in hippocampal neurons can lead to beneficial effects on neuroplasticity [14], while excessive activation of ionotropic GluRs results in deleterious effects [16,17]. In addition, spine morphology is also regulated by the same synaptic proteins that regulate actin and microtubules in the dendrite [18]. For example, the post synaptic density (PSD) is enlarged due to an increase in PSD protein95 (PSD95), which facilitates an early structural enlargement of dendritic spines [18,19]. BDNF-induced increase in PSD95 leads to microtubule rearrangements during the maturation period of dendritic spines [19]. Additionally, an increase in polysialylated-neural cell adhesion molecules promotes synaptic remodeling during persistent types of plasticity [20].

Functional plasticity is commonly interchanged with “synaptic plasticity” due to its primary involvement in synaptic transmission [21,22,23,24]. Synaptic plasticity may operate in the following ways: (1) growth of new synaptic connections or pruning of existing ones, (2) modification of the strength or efficacy of synaptic transmission, and (3) modulation of the excitability of existing synapses. Due to the diversity of neuronal functions, many forms of synaptic plasticity have been described, such as post-tetanic potentiation (PTP), long-term potentiation (LTP), and long-term depression (LTD) [24]. During synaptic potentiation, receptors are translocated to the post-synaptic membrane where they interact with other members of the PSD; however, during synaptic depression, the receptors are endocytosed [20]. Various signaling pathways (such as mitogen-activated protein kinases [MAPK] pathway, Wnt pathway, and BDNF-TrkB signaling) influence the induction of activity-dependent synaptic plasticity [25,26,27]. Such activity-dependent synaptic plasticity has been studied in experimental setups, where synaptic plasticity was induced through high- or low-frequency stimulation of neuronal afferents that cause LTP [28,29] and LTD [30] during synaptic transmission. The interplay of structural and functional plasticity is evidenced by synaptic plasticity that leads to structural modifications in dendritic spines; induction of LTP that promotes spine head growth, formation, and maintenance. However, LTD causes the spine heads to shrink and retract [31]. This relationship between structural and functional plasticity underlies vital processes that maintain the normal anatomy and physiology of the brain.

Volumetric and morphological changes in dendritic structures are widely studied in various physiological and pathological conditions [32,33]. Regulation of neuroplasticity in dendrites, spines, and synapses are sensitive to both positive (learning [34] and environmental enrichment [35]) and negative stimuli (radiation [36,37], stress [38], chemical exposure [39], aging [40,41,42] and neurodegenerative disease [43,44]). Moreover, neurodegenerative diseases, in particular, are described by dysregulations in the structural plasticity [45,46]. Dendritic and spine changes caused due to acute or chronic disturbances in brain tissue homeostasis may form the basis of neurodegenerative diseases [47]. The involvement of neuroplasticity in neurodegenerative diseases has been widely studied [45,48,49,50], as these changes in dendritic and spine structure in several brain regions serve as mechanisms for motor and non-motor features of various neurodegenerative diseases [51,52,53,54]. Additionally, aging is considered as a primary risk factor for the development of neurodegenerative diseases [55]; in fact, several review articles have already described dendritic structural alterations in the context of aging [56,57,58].

The hippocampus is an important brain region for its impressive capacity for lifelong plasticity [59]. Structural plasticity in the hippocampus is susceptible to a wide range of stimuli, many of which appear to have long-term impacts. For example, the rapid formation of new spines and enlargement of existing spines in hippocampal slices are associated with LTP-mediated increase in synaptic strength [9], whereas loss of post-synaptic actin and spine shrinkage are associated with LTD-induced actin depolymerization [60]. Furthermore, hippocampal neurons readily respond to environmental cues by showing differential pattern changes in dendritic and spine morphology [61]. The hippocampus is one of the brain areas that shows neuronal remodeling under several neurodegenerative diseases, including Alzheimer’s disease (AD), Parkinson’s disease (PD), Huntington’s disease (HD), multiple sclerosis (MS), vascular dementia (VD), frontotemporal dementia (FTD), and amyotrophic lateral sclerosis (ALS) [62,63,64,65,66,67].

AD, PD, and HD are highly prevalent neurodegenerative diseases [68,69], while MS is a non-traumatic, neuroinflammatory, and neurodegenerative disease affecting young adults [70]. VD and FTD are forms of dementia, which is a degenerative disease primarily characterized by intellectual and cognitive impairment [66]. ALS is a neurodegenerative disease with varying degrees of impairment of motor function and cognitive profile [71,72]. To date, numerous therapeutic approaches have been developed to relieve clinical symptoms in these diseases. However, non-motor dysfunctions respond poorly to conventional therapeutics and affect the quality of life (QOL) of the patients [73,74,75]. As a result, there is a need for improvement in the existing treatments or the development of entirely new strategies. The hippocampus has been the focus of neuroplasticity research for common neurodegenerative diseases, including AD, PD, HD, MS, VD, FTD, and ALS since it is a principal brain region for cognitive and emotional functions [76,77,78,79,80]. Several reviews [81,82,83] have already explored the involvement of functional or synaptic plasticity of the hippocampus in neurodegenerative diseases. Thus, in this review, we primarily focused on the role of structural plasticity of the hippocampus in neurodegenerative diseases.

In this study, articles describing structural plasticity of the hippocampus in neurodegenerative diseases, including AD, PD, HD, MS, VD, FTD, and ALS, were collected through a literature search using Google Scholar, PubMed, Web of Science, and Scopus. This review provides an overview of how structural plasticity in the hippocampus is involved in clinical and preclinical studies of neurodegenerative diseases. Further, this review suggests possible therapeutic approaches to regulate altered hippocampal neuroplasticity in neurodegenerative diseases. Additionally, the prevailing gaps in the knowledge of hippocampal neuroplasticity in neurodegenerative diseases will also be revealed to serve as starting points for future studies.

## 2. Hippocampal Dysfunction in Neurodegenerative Diseases

### 2.1. Alzheimer’s Disease

AD is one of the most prevalent neurodegenerative diseases with major neuropathological hallmarks as follows: (1) intra-neuronal accumulation of neurofibrillary tangles (NFTs) as a consequence of abnormal hyperphosphorylation of Tau (HPtau) proteins, (2) extracellular deposition of amyloid-β (Aβ) protein as senile plaques, and (3) massive neuronal death [84]. AD is an age-related disease featuring progressive loss of memory and cognitive abilities [85]. Individuals with AD usually undergo difficulties in learning, performance speed, recall accuracy, and problem-solving [86]. Hippocampal involvement in AD pathogenesis is evident from a wide range of clinical and preclinical studies [82,87,88,89,90,91]. In AD, the hippocampal cornu ammonis (CA) 1 subregion is the first target of pathological hallmarks (i.e., NFTs), followed by the subiculum, CA2, CA3, and dentate gyrus (DG) [92,93,94]. Particularly, overall neuronal loss in the CA1 region was predominantly observed in most of the studies that included patients with mild to severe AD [95,96,97]. Furthermore, impaired functional connectivity of the hippocampus with several other brain regions results in a combination of neuropsychological abnormalities in AD [80].

**Figure 1:**
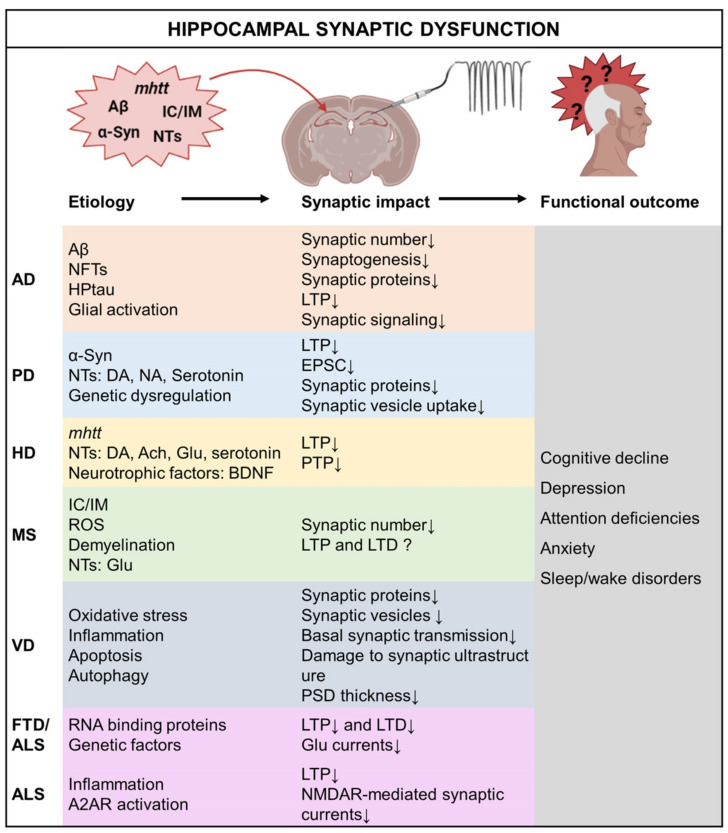
Schematic flow diagram of the correlation between etiopathogenesis and hippocampal synaptic dysfunction in neurodegenerative diseases. In neurodegenerative diseases, several etiological factors impact hippocampal synaptic plasticity, leading to functional outcomes such as cognition and emotional dysregulations. Abbreviations: Ach, acetylcholine; A2AR, adenosine A_2A_ receptor; AD, Alzheimer’s disease; α-Syn, alpha-synuclein; Aβ, amyloid beta; ALS, amyotrophic lateral sclerosis; BDNF, brain-derived neurotrophic factor; DA, dopamine; EPSC, excitatory post-synaptic current; FTD, frontotemporal dementia; Glu, glutamate; HD, Huntington’s disease; HPtau, hyperphosphorylated Tau; IC, inflammatory cells; IM, inflammatory mediators; LTD, long term depression; LTP, long term potentiation; *mhtt*, mutant-huntingtin; MS, multiple sclerosis; NA, noradrenaline; NFTs, neurofibrillary tangles; NMDAR, N-methyl-D-aspartate receptors; NTs, neurotransmitter systems; PD, Parkinson’s disease; PSD, post-synaptic density; PTP, post-tetanic potentiation; ROS, reactive oxygen species; VD, vascular dementia.

Cognitive impairment in early AD is largely due to synaptic dysfunction [98] (Figure 1). Causes of synaptic dysfunction in AD include decrease in overall cell count [99], synaptic deterioration due to soluble Aβ [100], synaptic pruning due to glial involvement [101], and significant decrease in synaptic vesicles [102,103]. Synaptic failure in the hippocampus is established in patients with AD as well as animal models [98,104]. In fact, hippocampal synaptic dysfunction is an early pathological hallmark in AD and is the underlying reason for memory impairment [105]. Hippocampal synaptic dysfunction associated with AD has been reported as decreased synaptic number [106,107,108], impaired synapse regeneration/synaptogenesis [109,110], loss of synaptic proteins (i.e., synaptophysin, synaptogyrin, synaptotagmin, syntaxin 1, PSD95, and Homer-1) [103,109,111,112,113,114,115], impaired LTP [108,111,113,114,116,117], and impaired synaptic signaling cascades (i.e., MAPK signaling and Wnt signaling) [118,119]. Memory impairment begins with subtle changes in hippocampus synaptic efficacy, before progressing to severe neuronal loss [120]. Interestingly, the synaptic dysfunction appears before the Aβ plaque formation, implying that a physiological deficit underlies the initial development of the disease [121,122,123].

### 2.2. Parkinson’s Disease

PD is the second most prevalent neurodegenerative disease in the world [124,125,126]. PD results from the deterioration of dopaminergic (DA) neurons in the extrapyramidal tract of the midbrain, along with the accumulation of alpha-synuclein (α-Syn) proteins (namely, Lewy bodies) in the nervous system. PD is a neurodegenerative condition known for both motor and non-motor symptoms [127]. Since the hallmark of PD is DA injury, much attention has been given to the structural, molecular, and functional changes in the nigrostriatal system. However, non-motor symptoms of PD that are associated with the hippocampus also significantly affect the QOL of the patients [128]. These symptoms include PD dementia, cognitive deficits, and sleep-wake disorders, which are evident in both the early phase and advanced stage of the PD [129,130]. Other non-motor symptoms including hyposmia, rapid eye movement, sleep behavior disorder, constipation, sadness, anxiety, depression, and apathy, may also occur before the beginning of motor deficits [131,132]. Several researchers suggest a key interaction between DA transmission and hippocampal neuroplasticity in non-motor symptoms; however, the complicated mechanisms underlying these symptoms may also involve non-DA systems. For example, hippocampal serotonergic [133,134] and noradrenergic (NA) [135] dysfunctions are reported in emotional dysregulation of PD.

Synaptic mechanisms underlying cognitive impairment in PD are widely reported in preclinical studies (Figure 1). For instance, impaired hippocampal synaptic plasticity in terms of decreased LTP is reported in neurotoxic models of PD, such as 6-hydroxydopamine (6-OHDA)-lesioned rats [136,137,138] and 1-methyl-4-phenyl-1,2,3,6-tetrahydropyridine (MPTP)-lesioned mice [136,137,139]. Similarly, α-Syn transgenic (TG) mice also showed reduced hippocampal CA1 LTP [136]. Hippocampal neurons exhibited reduced excitatory post-synaptic current (EPSC) [140] and progressive impairments in neuronal excitability [100] upon α-Syn preformed fibrils (PFF) treatment. Synaptic protein alterations in CA1 [136,137] and DG regions [138] are also reported in 6-OHDA-lesioned rats and PFF-treated primary cultured hippocampal neurons [100]. Several studies suggest the importance of DA in the maintenance of hippocampal LTP through regulation of dopamine D1/5 receptors [141,142,143] and stimulation of synaptic protein synthesis [144,145], both of which demonstrate an interplay between the DA input and hippocampal synaptic plasticity for the maintenance of hippocampus-related functions. It has been reported that L-DOPA, also known as levodopa and l-3,4-dihydroxyphenylalanine, restored hippocampal LTP and alleviated cognitive impairments in clinical [146] and preclinical [129,136] conditions of PD. Although aberrations in functional plasticity are often attributed to DA signaling, other neurotransmitters such as NA [138,147] and serotonin [148] have also been implicated. Recent advances in studies exploring the genetic basis of PD-highlighted genes that may be related to the disruptions in synaptic vesicle endocytosis, and act as significant contributors to its pathogenesis. These endocytosis disruption-related genes include auxilin (*DNAJC6*) synaptojanin 1 (*SYNJ1*), endophilin A1 (*SH3GL2*), leucine-rich repeat kinase 2 (*LRRK2*), parkin (*PRKN*), and vacuolar protein sorting ortholog 35 (*VPS35*) [44].

### 2.3. Huntington’s Disease

HD is a progressive autosomal dominant neurodegenerative disorder, which is characterized by a combination of progressive motor and cognitive impairments, as well as neuropsychiatric symptoms [149,150,151]. These symptoms include distinct behavioral changes, including chorea, dystonia, cognitive decline, and emotional impairments [137,152]. HD results from expanded three nucleotide (Cytosine, Adenine, and Guanine)-repeats in the *huntingtin* gene [153]. The neuropathological changes in HD include atrophy and cell loss in the caudate putamen of the striatum, where medium spiny neurons are the most vulnerable [154,155,156]. This has led to the striatum being the center of attention for molecular, structural, and functional studies of HD [157,158,159]. However, other brain regions such as the hippocampus are also affected in HD [160]. Non-motor symptoms in preclinical and clinical HD conditions are significantly related to the hippocampus [78,161]. Mild cognitive impairments have been detected in patients with HD long before (up to 15 years) the formal diagnosis based on the motor symptoms [162]. Additionally, deficits in episodic memory, processing speed, executive function, and visuospatial perception were found in the clinical stages of HD [68]. Studies using environmental and pharmacological interventions imply that basal serotonergic disruption in the hippocampus is a causal factor in depressive-like behavior in HD [78]. Moreover, altered extracellular glutamate (Glu) dynamics in the hippocampus may also underpin cognitive deficits in HD [163].

Different forms of functional plasticity are impaired in human patients and mouse models of HD [164,165,166,167,168,169,170,171,172,173,174]. These abnormalities in synaptic function include both short- and long-term plasticity (Figure 1). For instance, impairments in long-term plasticity in terms of deficient induction and maintenance of LTP, as well as impairments in short-term plasticity in terms of decreased PTP [164,175,176] are reported in HD. Disruption of LTP at the hippocampal CA3-CA1 synapse is reported in Hu97/18-TG [171], proline-rich tyrosine kinase 2 (Pyk2)-deficient [172], and Q175FDN-knock-in mice [176]. LTP mediated by the N-methyl-D-aspartate receptors (NMDARs) at CA1 synapse in the hippocampus is also impaired in Pyk2-deficient [172], R6/2-TG [165], and R6/1-TG mice [177] at the symptomatic stage. These impairments are known to underlie cognitive disabilities, such as learning and memory [24,178,179]. Long-term deficits in the hippocampus are likely to be mediated by disturbances in a range of signaling pathways, contributing to the hippocampal dysfunction in HD [176]. Other possible factors mediating synaptic plasticity in HD are neurotransmitters such as DA, acetylcholine (Ach), and Glu [180], and neurotrophic factors such as BDNF [181].

### 2.4. Multiple Sclerosis

MS is a progressive autoimmune, neuroinflammatory, and neurodegenerative disease of the central nervous system (CNS), resulting from an autoimmune attack on myelinated axons [182]. This autoimmune attack induces inflammation, which leads to subsequent oligodendrocyte damage and demyelination in the CNS [183,184]. MS is a non-traumatic disabling disease affecting young adults [70]. The pathological hallmark of MS is perivascular inflammatory lesions that lead to demyelinating plaques [183]. The bulk of the inflammatory infiltrate is comprised of T-lymphocytes (dominated by major histocompatibility complex class 1 restricted CD8^+^ T-lymphocytes), along with much lower numbers of B-cells and plasma cells [185]. Hippocampal involvement, as evident in many patients with MS, is characterized by demyelination, neuronal damage, and synaptic abnormalities. Hippocampal demyelination is common and extensive in MS [186,187,188]. Cognitive decline, which occurs even in the absence of motor impairment, is also a hallmark of MS [189,190]. Patients with MS often show long-term memory impairments [191], attention deficiencies [192], and reduced information processing speeds [193]. Among these non-motor aspects of MS, depression, memory impairment, and psychosis are associated with hippocampal dysfunction [194] and disconnection from multiple brain networks [195].

Functional synaptic plasticity seems to be altered during the disease progression of MS [196] (Figure 1). Synaptic plasticity is disrupted in patients with MS, suggesting a synaptic basis for the cognitive abnormalities linked with this disorder [197,198]. Demyelinated hippocampi from patients with MS demonstrated a negative impact on the molecules involved in axonal transport, synaptic integrity and plasticity, Glu homeostasis, and learning and memory [188]. Demyelinated areas in the hippocampus also exhibited a significant decline in the number of synapses [188,199]. Differential outcomes on hippocampal synaptic plasticity in MS have been reported in a number of investigations. For instance, impaired hippocampal LTP and cognitive behavior are evident in both early [200,201,202] and late phase [201,202,203,204] of experimental autoimmune encephalomyelitis (EAE) mice, an animal model for MS. This impairment of LTP is associated with an altered assembly of NMDARs and a selective increase of interleukin-1β (IL-1β) in the hippocampus [200]. In contrast, increased LTP and blocked LTD are reported in the hippocampal CA1 region in EAE mice in the late phase [205]. These disparities may be attributed to the differences in EAE-inducted conditions, utilized animal, electrophysiological methods, and clinical severity at the time of evaluation, all of which interfere with the interpretation and comparability of the research [203,206].

During the past decades, many studies have focused on replicating and understanding the pathophysiological mechanisms underlying the altered synaptic plasticity in MS. For example, Di Filippo et al. [206] reviewed several mechanisms that influence synaptic plasticity during the course of MS using the EAE animal model. Inflammatory cells, including invading T lymphocytes [207], activated microglia [200,207], and some cytokines, such as IL-1β [200], play important roles in fluctuation in LTP and alterations in synaptic plasticity in EAE [207]. Particularly, LTP impairment is related to strong activation of the hippocampal microglia and elevated levels of IL-1β during the first acute relapse of relapsing-remitting EAE mice [200] and monophasic EAE rats [208]. IL-1β regulates the expression of ionotropic receptors such as NMDA [209,210] and α-amino-3-hydroxy-5-methyl-4-isoxazolepropionic acid receptors (AMPAR) [211], which are important in synaptic function and LTP induction and maintenance. Interestingly, the altered hippocampal synaptic plasticity (either enhanced or impaired) in the remitting phase of EAE is associated with persistent glial activation and elevated levels of IL-1β and nicotinamide adenine dinucleotide phosphate oxidase, a reactive oxygen species (ROS) producing enzyme [202,205,212]. Collectively, these findings imply that hippocampal LTP in EAE, and thus, in MS, is a dynamic process that is underlined by inflammatory signaling and neurotransmission (Figure 1). The onset of disturbance in LTP is debatable, although most studies demonstrate onset during late-phase rather than the early phase. A predominant hypothesis is that in comparison with the less severe early phase of the disease progression, the late phase exacerbates axonal loss, gliosis, and tissue degeneration [203,213].

### 2.5. Other Neurodegenerative Diseases

Apart from the above-mentioned major neurodegenerative diseases, other neurodegenerative conditions are also reported to involve the hippocampus. Both VD and FTD involve hippocampal dysfunction at variable levels [214,215]. VD is caused by cerebrovascular changes leading to cognitive impairment [216,217]. FTD is a clinically, pathologically, and genetically heterogeneous neurodegenerative disease [218]. The involvement of the hippocampus in FTD has only recently been explored [219]. Another neurodegenerative disease that affects multiple brain regions including frontotemporal, subcortical, and cerebellar regions, is ALS. Originally being a motor neuron disease [71], the hippocampal dysfunction in ALS has only recently been recognized along with memory deficits [72,220].

Synaptic dysfunction in VD has been reported in terms of decreased synaptic proteins [221,222,223], synaptic vesicles [224], and LTP [224,225] (Figure 1). In rat models of VD with bilateral common carotid artery occlusion (BCCAO), impaired basal synaptic transmission [225] and damaged synaptic ultrastructure [222] were observed. Most plasticity changes were alleviated with antioxidative therapies [65,225], supporting the strong correlation between hippocampal synaptic dysfunction and oxidative stress in VD [226]. FTD and ALS are discussed for their common pathogenic mechanisms that have different outcomes [227]. Therefore, several genetic models are subjected to experiment the common pathological outcomes in ALS and FTD. Disruption of hippocampal synaptic plasticity is reported in *C9orf72*-deficient mice in terms of reduced LTP and LTD [228]. Furthermore, amplitude of the Glu currents were reduced without changing the paired pulse in the hippocampal slices from UBQLN2P497H TG mice [227]. Although ALS is primarily a motor neuron disease, it is now being recognized as a multi-system condition with disease-specific patterns of hippocampal pathology [229]. The evidence for memory deficits related to hippocampal dysfunction is widely found in ALS [229,230,231,232]. Decreased functional connectivity among bilateral hippocampus, bilateral para hippocampal gyri, and cerebellum was revealed in patients with ALS [231,233]. In SOD1G93A TG mice, a genetic model for ALS, LTP impairment and decreased NMDAR-mediated synaptic currents were associated with the symptomatic stages. However, in the pre-symptomatic stage, the pre-synaptic function was enhanced, while increased adenosine A_2A_ receptor levels were found at both symptomatic and pre-symptomatic stages [234]. Similarly, increased LTP in pre-symptomatic SOD1G93A TG mice was observed along with higher expression of GluR1 [235].

Chronic cerebral hypoperfusion due to various reasons results in glucose and oxygen depletion in the CNS, leading to the pathophysiological process of VD (Figure 1), which includes oxidative stress, inflammatory response, cell apoptosis, autophagy, and synaptic damage [236,237,238,239]. Although the precise mechanism for hippocampal synaptic dysfunction is unclear, the previously mentioned pathophysiological processes are suggested as the primary causes for the synaptic ultrastructural changes, synaptic loss, and reduction in PSD thickness [224]. Furthermore, the influence of VD-induced cholinergic deficits on LTP impairment has been described [240,241,242]. Hippocampal synaptic function in SOD1G93A TG mice, a model of ALS, is largely associated with neurotransmitter imbalances (Figure 1). For example, increased LTP in pre-symptomatic SOD1G93A TG mice was observed together with higher expression of GluR1 [235] and adenosine A_2A_ receptor levels [234]. Adenosine A_2A_ receptor activation enhances neuronal excitability, resulting in excitotoxic and neuroinflammatory consequences, both of which are hallmarks of ALS [243]. However, the observation of higher adenosine A_2A_ receptor levels even at the symptomatic stage of SOD1G93A TG mice [234] with impaired LTP is still unexplained. In contrast, the NMDAR dysfunction and LTP impairment are possibly a result of an interplay between neurotransmitter systems; NMDARs are under the control of adenosine A_2A_ receptors, during synaptic activity regulation [244]. ALS and FTD are neuropathologically related by the RNA-binding protein TDP-43 (TAR DNA-binding protein) [245]. TDP-43 is involved in the splicing, transport, and stability of several mRNAs which encode proteins playing important roles in synaptic function [246,247,248]. Thus, the genetic regulation of hippocampal synaptic plasticity in ALS and FTD cannot be excluded (Figure 1). However, hippocampal synaptic alterations in FTD are still poorly documented and need further investigation.

## 3. Structural Plasticity in the Hippocampus: General Overview

### 3.1. Dendritic Complexity

The hippocampus harbors a heterogeneous population of cells distributed in subfields DG, CA1, CA2, and CA3 [249,250]. Pyramidal neurons in the CA region consist of apical dendrites, which rise from the upper pole of the cell body, and basal dendrites, which emerge from the base of the soma to form the basal dendritic arbors [251]. The granule cells of the DG region consist of a cone-shaped apical dendritic tree emerging from an elliptical cell body [252]. Dendritic morphogenesis is a well-organized, but a complicated process that includes the formation of dendritic branches and spines, which facilitates the connection of neurons [253,254].

Dendritic arbors are highly dynamic structures that expand and retract in response to stimuli and are stabilized and maintained by post-synaptic signaling [255,256]. Structural dynamics of dendrites in normal, as well as diseased brain, include active growth and turnover of dendritic branches [256]. Such alterations in the dendritic structure include changes to the dendritic branching pattern, fragmentation of dendrites, and retraction of dendritic branches, all of which are associated with the reorganization of the neuronal cytoskeleton [257]. Microtubule dynamics play a key role in shaping the dendritic arbors. Dendrite branching and microtubule dynamics are regulated through a large number of cellular factors, including microtubule regulatory proteins [258], neurotransmitters [259], glucocorticoids [260], and various growth factors [261]. Thus, changes in any of these factors can result in alterations of the dendritic arbors, as evident in many neurological conditions [262,263,264,265].

### 3.2. Dendritic Spine

Hippocampal pyramidal and granule cells bear dendritic spines with minor differences in neck diameter, spine length, spine volume, head volume, and PSD area [266]. In general, dendritic spines are tiny, actin-rich, and specialized structures, which arise from the neuronal dendrites [267]. These membrane protrusions on the shaft of dendrites form the post-synaptic component of the excitatory synapse, and their structure and density are important factors in the functional plasticity [268]. Dendritic spines are morphologically categorized as mushroom, thin, stubby, filopodium, and branched [269,270]. Among them, mushroom spines are most stable and make functionally stronger synapses, which are responsible for the memory storage [271]. However, thin spines with a smaller head and narrow neck are relatively less stable [31]. Stubby spines without an obvious constriction between the head and shaft appear more stable and persistent than thin spines [272]. Filopodium spines are hair-like [273] and often short-lived [274], but are important for the rapid selecting potential of synaptic partners before a mature synapse is established [274]. Branched spines consist of multiple head regions, where each head synapses with a different pre-synaptic axon [275].

Overall, dendritic spines consist of a major component of the synapses between hippocampal neurons [276] and undergo structural plasticity in terms of formation, shedding expansion, and atrophy [277]. Rapid modifications in dendritic spines are also involved in rearrangements of the actin cytoskeleton [266]. Thus, dendritic spines serve as primary locations for synaptic function, and their disturbances can alter spine morphology and functions, as observed in normal aging as well as many neurodegenerative diseases [278].

## 4. Hippocampal Structural Plasticity in Neurodegenerative Diseases

Table 1 summarizes recent evidence regarding alterations of structural plasticity in the hippocampus in neurodegenerative diseases, including AD, PD, HD, MS, VD, FTD, and ALS.

### 4.1. Structural Plasticity in the Hippocampus with AD

#### 4.1.1. Dendritic Complexity

Dendritic abnormalities are common in AD and appear early in the disease, which includes dystrophic neurites and decreased dendritic complexity [279]. A wide range of studies exploring the molecular and genetic mechanisms of AD established that molecular lesions in the asymptomatic phase are the early events that lead to neuronal damage and cognitive decline in the symptomatic phase [304,305]. Therein, Aβ aggregation and HPtau are accompanied by morphological changes, such as changes in neuronal shape, volume, and complexity. To this end, decreased dendritic length and dendritic arborization were observed in the hippocampi of patients with AD [279]. In experimental models of AD, dendritic complexity was demonstrated to be reduced in the hippocampi of amyloid precursor protein/presenilin 1 (APP/PS1)-TG mice [114,277,280], apolipoprotein E4 (APOE4)-TG mice [281], and primary cultured hippocampal neurons of miR-34c-transfected mice [282]. In contrast, an increase in dendritic complexity was reported in the N-tau-TG mice [283]. However, in TgCRND8 mice, no changes were observed in the dendritic arborization [112]. The variability in the observations may be due to differences in the animal models and the parameters used for selecting the neurons to be included in the studies (e.g., absence or presence of nearby Aβ plaques/NFTs).

The involvement of Aβ aggregations and HPtau in altered dendritic arborization in AD is well documented [277,306]. Aβ peptides within or near neurites are directly involved in synaptic disruption through mechanical factors, including mitochondrial dysfunction, oxidative stress, and calcium dysregulation [307,308]. A significantly high percentage of dendrites ending within and adjacent to NFTs are often reported to appear atrophic, which indicates substantial disruption of the cytoskeleton [277]. Another hypothesis suggests that fibrillary amyloid is detrimental, as they cause local structural disruptions and eventual neurite breakage [277]. Tau can bind to tubulin via its microtubule-binding domains [309] and regulate the microtubule dynamics. Deficits in tau protein led to reduced neuronal outgrowth disrupted axonal extension, and reduced microtubule density [310]. In AD, HPtau detaches from microtubules and aggregates into NFTs [306], which impairs the normal microtubule structure in the dendrites. However, rather than an individual effect, Aβ aggregates and HPtau interact to develop dendritic pathologies [306].

#### 4.1.2. Dendritic Spine Density and Morphology

In general, reduced spine density and altered spine morphology are observed before the onset of reduction in neuronal density and synapses in the genetic models of AD. This is in corroboration with the impaired dendritic spine density reported in patients with AD [280]. Significant decrease in spine density was evident in APP/PS1-TG mice [114,280], APOE4-TG mice [281], CRND8-TG mice [112], 2576-TG mice [116], Aβ-infused rats [113,115], miR-34c-transfected mouse primary cultured hippocampal neurons [282], and hippocampal slices of Aβ-treated rats [117]. In contrast, an increase in spine density was reported in N-tau-TG mice [283]. Reduction in spine length was also noted in APP/PS1-TG mice [284]. Moreover, filopodia or thin dendritic spines were reduced in the miR-34c-transfected mouse primary cultured hippocampal neurons [282], CRND8-TG mice [112], and Aβ-infused rats [115]. On the other hand, a decrease in mushroom spines was reported in APP/PS1-TG [114] and APP-knock-in [285] mice.

There are several suggestions regarding spine loss in AD. It is hypothesized that mainly soluble, not fibrillary Aβ contributes to synaptic dysfunction and spine loss, which occur before plaque and tangle formation, and eventually lead to cognitive dysfunction [311]. Synapse loss is the most powerful pathological correlate of dementia found in the AD [50]. Aβ mediated synaptic failure is assumed to be caused by slightly high post-synaptic calcium concentrations and AMPAR elimination, which would reduce spine formation [312]. In addition, several studies have suggested that Glu circulation and GluR alterations due to Aβ pathology would result in spine pruning [112,115]. However, the underpinning molecular mechanisms remain uncertain. One of the proposed mechanisms is that Aβ might influence dendritic spines through the serum-inducible kinase-spine-associated rap guanosine triphosphatase-activating protein signaling pathway by influencing the synaptic integrity [313]. In addition, recent studies have considered tau proteins as key mediators of Aβ induced synaptic dysfunction and loss [311]. Several kinases, including cyclin-dependent kinase 5, Fyn, glycogen synthase kinase-3 (GSK3), and MAPK, can be inappropriately activated by Aβ, resulting in HPtau [314]. HPtau allows Fyn to be targeted to the PSD of dendritic spines, where it phosphorylates GluN2 and stabilizes its association with PSD95, increasing the excitotoxicity [278]. Additionally, detachment of HPtau from microtubules and its aggregations into NFTs can directly affect the actin cytoskeleton of spines [306]. Collectively, spine alterations in AD are mediated by the interplay of several factors including pathologic protein deposits, neurotransmitters, and kinases. Future studies are warranted to help elucidate the exact mechanism of this interaction. The mechanisms underlying the current AD therapy [315,316,317], which target these abnormal proteins, can be connected to the ability to rescue impairment of structural plasticity in the hippocampus with AD. Future understanding of molecular mechanisms responsible for this interplay may expose novel therapeutic targets as well as increase the effectiveness of existing anti-HPtau and Aβ therapies.

### 4.2. Structural Plasticity in the Hippocampus with PD

#### 4.2.1. Dendritic Complexity

A wide range of clinical and preclinical studies of Parkinsonism have revealed dystrophic alterations in the dendrites of striatal medium spiny neurons (MSNs) [318,319,320,321,322]. However, the evidence for structural plasticity in the parkinsonian hippocampus is limited. Only a few studies have investigated hippocampal structural plasticity in animal models for PD. To the best of our knowledge, there are no such reports from clinical studies. Dendritic length and branching were reduced in primary cultured hippocampal neurons [286] and newly generated DG neurons [287,288] in the LRRK2-mutant mice. However, a previous study showed that the dendritic complexity of both CA1 and DG subregions was not altered in the MPTP-lesioned mice [289]. Therefore, further studies are needed to confirm dendritic pathology in PD animal models, since variations in observations due to different models cannot be neglected.

A previous study hypothesized that LRRK2 mutation impairs rearrangement of actin cytoskeleton in neuronal morphogenesis of the hippocampus; however, relevant mechanisms are yet to be fully elucidated [288]. In this regard, hippocampal dendritic arbors need to be investigated in PD. First, similar to the observed striatal dendritic pathology, DA is also important in the modulation of hippocampus-dependent learning and memory [323], thus interactions between DA depletion and hippocampal dysfunction in PD is highly likely [129,324]. Dopamine D1 receptors play a prominent role in synaptic plasticity and learning and memory in the hippocampus [325]. D1 receptors-mediated dendritic growth is reported in the medial prefrontal cortex [326], although it is not yet tested in the hippocampus. Second, α-Syn pathology, a hallmark of Lewy neurites in sporadic PD, impairs dendritic branching in adult-born granule cells [327] and midbrain neurons [328]. Histological evaluations revealed α-Syn as the main component that leads to axonal and dendritic pathologies in the hippocampus [329]. However, the possible effects of α-Syn pathology on dendritic arbors in the hippocampus need further clarifications using clinical and preclinical models of PD. Additionally, hippocampal serotonin and NA deficiencies were noted in several animal models for PD [148,330,331], and both serotonin and NA were involved in the regulation of the neurite extension [332]. Collectively, these findings may reveal possible consequences on hippocampal dendritic pathologies in PD.

#### 4.2.2. Dendritic Spine Density and Morphology

In general, decreased spine density and altered morphology are described in hippocampal neurons in PD models. Spine density in the hippocampal DG region was decreased in MPTP-lesioned mice along with a remarkable decrease in the proteins that mediated neuroplasticity mechanisms and impaired cognitive function [139]. Similarly, a recent study found decreased spine density in both CA1 and DG subregions of the MPTP-lesioned mice during the late phase of PD [289]. In LRRK2-mutant mice, newly generated DG neurons were found with reduced dendritic spines [288]. Moreover, PFF-treated primary cultured hippocampal neurons exhibited reductions in dendritic spine density and head diameter of mushroom-shaped spines [290,291]. These observations suggest that the pathology of PD is not merely restricted to the nigrostriatal pathway.

There are several possible factors underlying the spine pathologies in hippocampal neurons in the PD brain, such as alterations in neurotransmission (i.e., DA, serotonergic, NA, and cholinergic systems) and Lewy bodies [333]. In mouse primary cultured hippocampal neurons, NA and DA agonists enhanced the spine density by means of elevated cAMP concentration [334], which suggests a possible mechanism underlying the spine pathology in DA-deprived hippocampi of PD. The DA levels decreased in the hippocampi of a PD animal model [324]. Similarly, deficiencies in serotonin and NA have been found in other PD animal models [148,330,331], both of which are also involved in the regulation of spine growth in hippocampal neurons [335].

Accumulation of α-Syn also occurs in the hippocampus [336]. Reductions in spine density have occurred only in wild-type neurons, but not in hippocampal neurons of α-Syn deficient mice, suggesting that the changes in spine morphology result from fibril-induced corruption of endogenously expressed α-Syn [290]. α-Syn, a protein abundant in the pre-synaptic terminals, has been postulated to polymerize purified tubulin into microtubules [337] and aid in vesicle fusion and recycling [338]. In PD, aberrant soluble oligomeric conformations of α-Syn mediate the disruption of cellular homeostasis and eventual death [339]. Additionally, α-Syn interacts with serotonin transporter activity by sequestrating it from the cellular membrane [340], which exerts deleterious effects on the structural plasticity [341]. Thus, dysregulations of dendritic spines in the hippocampus of the PD brain seem to be associated with interactions between several factors (neurotransmitters and abnormal protein aggregates), which contributes to the pathophysiology in both preclinical and clinical PD.

Observations from patients with PD suggest that neuronal changes in the hippocampus may trigger neuropsychological symptoms of PD [342]. Moreover, unlike the well-known motor functions, the neuropsychological symptoms have a relatively poor response to DA therapy, which further confirms the extranigral control of such symptoms [73]. Future studies on the alteration of neuronal architecture in the hippocampus may explain most of the unexplained pathophysiology of neuropsychological features in PD.

### 4.3. Structural Plasticity in the Hippocampus with HD

#### 4.3.1. Dendritic Complexity

Impairments in the dendritic complexity have been well documented in the MSNs of the striatum in animal models of HD [343,344,345]. However, dendritic structure in hippocampal neurons widely remains unknown in HD patients and animal models. A study using primary hippocampal neurons from R6/1-TG mice reported a decrease in neurite number and complexity [292]. However, the evidence was insufficient to draw conclusions about hippocampal dendritic changes in HD. Therefore, the description of hippocampal structural plasticity in HD pathogenesis requires further elucidation in both clinical and preclinical studies.

The pathology of aberrant dendritic structure in HD has been linked to the involvement of the mutant *huntingtin* gene (*mhtt*) [346]. In the normal brain, *htt* is an important regulator of mitochondrial function, whereas in the mutant background, *mhtt* causes several mitochondrial changes, such as loss of membrane potential and increased oxidative stress [346]. *mhtt* affects ATP levels in synapses [347] and leads to possible pathophysiological implications since several molecular processes in the synapses are susceptible to low ATP levels [348]. Hippocampal dendrite outgrowth is a highly energy-dependent process [349], which may be impaired when there is a reduction in mitochondrial ATP production [350]. Recent developments in metabolic research at the cellular level have provided critical insights into the cellular and molecular underpinnings of the connection between neural activity and energy consumption [351]. These techniques may help in finding methods for restoring ATP in synapses with *mhtt*-induced low ATP levels. Additionally, *mhtt* is also involved in the vesicular transport of neurotrophic molecules like BDNF along the microtubules [352], which are important for dendritic tree development. Ultimately, the functional capacity of the hippocampus is directly related to the complexity of neuronal dendritic arbors [353]. In addition, stimulation of prostaglandin E2 receptors increased the dendritic growth, recovered the neurochemical levels in R6/1-TG primary hippocampal neurons, and reduced *mhtt*-induced cognitive deficits in R6/1-TG mice, suggesting its therapeutic potential for HD [292].

#### 4.3.2. Dendritic Spine Density and Morphology

There are conflicting findings of alterations of dendritic spines in hippocampal neurons of the HD brain. Reduction of dendritic spine density in the CA1 subregion of the hippocampus has been reported in Hip14-deficient [293], R6/1-TG [294], and Pyk2-deficient [172] mice. However, in a different study that used R6/1-TG mice, dendritic spine density in the hippocampus remained unchanged [295]. These discrepancies in observations may be due to differences in the experimental setup and genetic background. Structural changes in hippocampal spines are frequently synonymous with impaired learning and memory in animal models of HD [172,292,293,294]. Therefore, the structural changes in hippocampal dendritic spines may have a role in the cognitive and neuropsychiatric deficits in HD.

The underlying mechanism for spine pathology in HD is still not clear. *mhtt* affects a variety of cellular and molecular processes that influence loss of spines and synaptic dysfunction, such as protein trafficking and aggregation, protein-protein interaction, calcium signaling, mitochondrial function, gene transcription, neurotransmitter release, and receptor activation, and neurotrophic support [347,354,355]. Among them, the mitochondrial function appears to be the predominant factor affecting spine maturation and morphogenesis [356]. For instance, in hippocampal neurons, manipulations that reduce dendritic mitochondrial content led to loss of synapses and dendritic spines [357]. *mhtt* impaired the mitochondrial transport in neuronal processes of the hippocampus of BACHD-TG mice [358], suggesting a possible mechanism involving spine pathologies in HD. Chronic inflammation involving glial activation is another possible pathway that can negatively impact hippocampal spine density in HD. For example, localized inflammatory response in the CA1 region and upregulation of nuclear factor kappa B (NF-κB) signaling have been observed in R6/1-TG mice along with reduced spine density [294]. Treatments to alleviate inflammatory responses also recovered these spine pathologies [359]. Thus, the use of anti-inflammatory pathways in maintaining spine structural integrity in HD is important.

### 4.4. Structural Plasticity in the Hippocampus with MS

#### 4.4.1. Dendritic Complexity

Aberrant neurite orientation dispersion in the spinal cord serves as a marker of microstructural pathology in patients with MS [360]. Extensive dendrite and neuronal soma atrophy have also been observed following axonal lesions in the spinal cord of patients with MS [361]. However, hippocampal dendritic pathologies in MS are also reported. In EAE mice at 20 days post-induction, dendritic length, and intersections were reduced in the molecular layer of the hippocampal DG [296,297]. These structural changes coexisted with hippocampus-dependent memory deficits in the mice [297].

In MS, there are several possible explanations for these structural modifications in the hippocampus. T-cell mediated autoimmunity and reactive gliosis trigger inflammatory mediators, including tumor necrosis factor α (TNFα) and interferon γ (IFNγ), which are toxic to oligodendrocytes [362,363,364] and inhibit neuronal precursor cell proliferation [365]. Defects in oligodendrocyte proliferation lead to hippocampal demyelination, which in turn leads to decreased dendritic arborization of hippocampal neurons [366]. The neurons in the CA1 region are highly vulnerable to a variety of stressors, including glutamate-mediated excitotoxicity [367], which has been implicated in the MS pathogenesis [368]. Another potential factor that may influence hippocampal neuroarchitecture is high-dose corticosteroids, which are routinely used to treat acute relapses. Corticosteroids significantly affect the CA1 neurons [369,370]. In fact, patients with MS, who are given large doses of steroids, experience transitory abnormalities in the declarative memory [371,372,373]. Thus, morphological changes in neurons of the hippocampus could reflect a variety of possible neuropathological processes, including demyelination, decreased dendritic density, and/or neuronal loss. However, the presently available evidence is too limited to attain a consensus regarding the role of hippocampal structural plasticity in MS.

#### 4.4.2. Dendritic Spine Density and Morphology

Dendritic spine alterations in the hippocampus have not been evaluated in patients with MS as well as EAE mice. Particularly, as fatality is rare in the early stage of MS, there is limited information about the acute effects of hippocampal demyelination on spine pathologies in patients with MS. However, there was no change in the spine density of demyelinated hippocampi of cuprizone-diet fed mice, except for an increase in the proportion of mushroom-shaped spines [298]. This increase was nullified with the withdrawal of the cuprizone diet and subsequent remyelination.

In the cuprizone-diet fed mice, the precise mechanism that links demyelination to dendritic spine abnormalities remains speculative. One possibility is that an inflammatory response via microglial activation may decrease the synaptic connectivity during the cuprizone-diet-induced demyelination [374]. Second, it has been shown that activated microglia secrete inflammatory cytokines and/or chemokines (i.e., IL-1β, TNFα, and IFNγ), which may also negatively impact neurogenesis and modulate dendritic spine morphology [375,376]. Third, as brain activity is a critical regulator of neuronal proliferation and synaptic connectivity [377,378], reduced brain activity due to demyelination can negatively impact hippocampal neurogenesis and spine densities. Nevertheless, the direct effect of cuprizone on proliferating cells and synaptic connectivity cannot be ignored [375]. However, the current findings from cuprizone-diet-induced demyelination in the hippocampus are not enough to infer its relevance to MS. Moreover, the demyelination in MS is more likely to occur over decades (chronic) in contrast to the acute demyelination mediated by the cuprizone diet. Therefore, a complete elucidation of the effect of demyelination on spine pathology in animal models with chronic hippocampal demyelination is warranted.

The association between microglial activation, demyelination, neurodegeneration, and disability has been established in patients with MS [379] as well as in EAE animal models [380]. Planche et al. [297] suggested a relationship between microglial activation, dendritic damages, synaptic plasticity disruption, and memory impairment, especially in the DG region. Despite being the most widely used model of MS, the specific spine pathologies in the hippocampus of EAE models remain to be elucidated.

### 4.5. Structural Plasticity in the Hippocampus with Other Neurodegenerative Diseases

#### 4.5.1. Dendritic Complexity

Total dendritic length, dendrite numbers, and dendrite crossings were significantly reduced in both CA1 and DG regions of rat models of VD with BCCAO [65,299]. Hippocampal dendritic branching and outgrowth were reduced in primary cultured rat hippocampal neurons with TDP-43 overexpression, as in an in vitro model of ALS/FTD [67]. In hSOD1G93A TG mice, aberrant dendritic complexity is readily reported in motor neurons of the primary motor cortex [381], pyramidal neurons of the medial prefrontal cortex [382], and lower motor neurons of the brainstem and spinal cord [383,384]. However, no changes were observed in the hippocampal dendritic complexity in the SOD1G93A TG mice [303].

Chronic brain hypoperfusion induces Aβ aggregation, HPtau, and cell death [385,386,387]. In VD rats, aberrant dendritic complexity in the hippocampus was associated with miR-195 mediated overproduction of N-terminal β-amyloid precursor protein (N-APP) [299], suggesting that miR-195 is a key link between the hallmarks of both AD and VD. It is well known that Aβ aggregation insults the dendritic complexity of hippocampal neurons, as described in Section 4.1.1. Therefore, reduction of hippocampal dendritic complexity in VD would share, at least in part, the same pathological mechanisms with AD. In addition, as one of the risk factors for VD, arterial hypertension has recently been reviewed as an altering factor of the hippocampal dendritic complexity in both human and animal models [388]. In a proposed model for ALS/FTD, dendritic growth is suppressed with the elevation of TDP-43 protein [67], primarily through RNA-binding functions that disrupt the functionality of many RNA transcripts. Furthermore, microtubule-associated tau (MAPT) is one of the three most common gene mutations in the FTD [389]. MAPT is critical in the formation and stabilization of axonal microtubules and neurite growth, and its mutation changes dendritic structure [390,391]. However, changes in hippocampal structural plasticity in ALS and FTD are still unclear due to the limited and contradictory literature arising from different animal models.

#### 4.5.2. Dendritic Spine Density and Morphology

In VD rats, densities of dendritic spines in both DG [65,300] and CA1 [301,302] regions of the hippocampus were decreased, largely due to the decrease in mushroom spines [65,301,305]. Moreover, expressions of spinophilin, a spine marker protein [301], and BDNF [302] were significantly lower in the CA1 region of VD rats. Similarly, with the major neurodegenerative diseases, aberrant hippocampal spine density was also associated with cognitive deficits in VD [301,302]. However, there was no difference in dendritic spine density in the DG region of the hippocampus of SOD1G93A TG mice, an animal model for ALS [303].

In general, cerebral hypoperfusion in VD animal models results in depleted ATP, mitochondrial dysfunction, and thereby increased ROS production and oxidative stress leading to a range of pathological events [392]. However, several molecules that play key roles in the major neurodegenerative diseases seem to take part in non-AD dementia as well. BDNF is one neurotrophic factor of such importance, which was found to be decreased in the VD hippocampus together with the suppression of the AMPK pathway [302]. Enhancement of BDNF levels and activation of the AMPK pathway in the hippocampus with VD simultaneously cured the spine pathology and cognitive deficits [302]. However, the role of the AMPK pathway in VD and other neurodegenerative diseases should be further studied, as there are contradictory reports on AMPK activation in neurodegenerative diseases, reporting worsening of the neuropathological and behavioral phenotypes [393,394]. Another highly plausible pathway, which could be involved in the spine pathology observed in VD, is GSK-3β and β-catenin signaling. GSK-3β activation has been noted in an animal model for VD in parallel with hippocampal spine density reduction [300]. Activation of GSK-3β has also been shown to increase the levels of pro-inflammatory cytokines, such as IL-1β and TNF-α [395], which further leads to detrimental effects on the dendritic structural plasticity [396,397]. Thus, the interplay of these proposed mechanisms needs further studies in order to clarify their role in the dendritic spine pathologies observed in VD. Moreover, alteration of spine density and morphology in other neurodegenerative diseases, such as FTD and ALS, are poorly documented and need further investigations.

**Figure 2:**
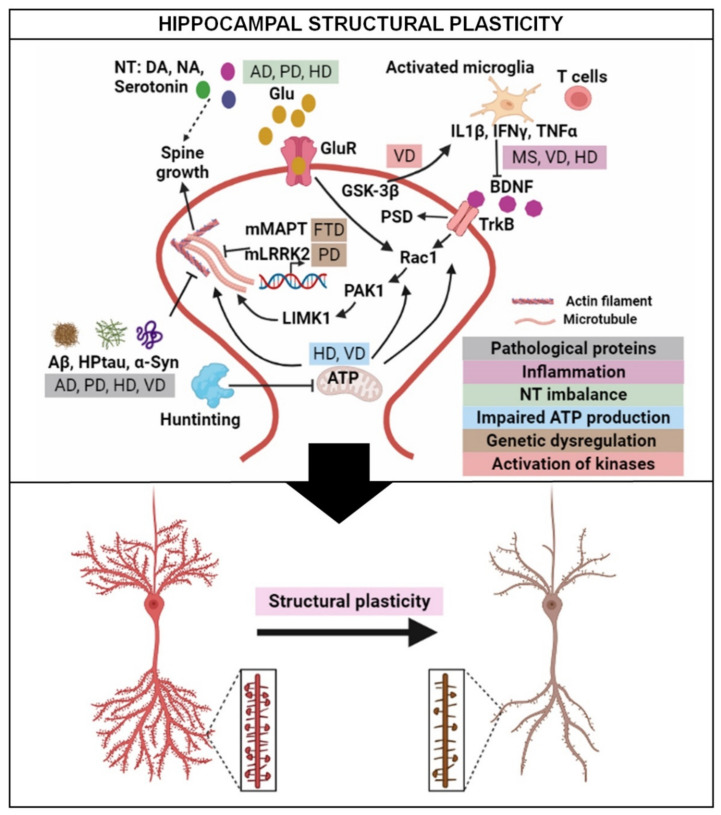
Schematic illustration of proposed mechanistic pathways for alteration of structural plasticity in the hippocampus in neurodegenerative diseases. During neurodegenerative diseases, pathological proteins, inflammation, neurotransmitter imbalance, impaired energy production, genetic factors, and activation of kinases affect the actin cytoskeleton and/or microtubule arrangement through different pathways. Abbreviations: AD, Alzheimer’s disease; α-Syn, alpha-synuclein; Aβ, amyloid-β; ATP, Adenosine 5′-triphosphate; BDNF, brain-derived neurotrophic factor; DA, dopamine; FTD, frontotemporal dementia; Glu, glutamate; GluR, glutamate receptor; GSK-3β, glycogen synthase kinase3β; HD, Huntington’s disease; IFNγ, interferon γ; IL-1β, interleukin 1β; LRRK2, leucine-rich repeat kinase 2; LIMK1, LIM kinase 1; MAPT, microtubule-associated tau; MS, multiple sclerosis; NA, noradrenaline; NT, neurotransmitter; PAK1, P21 (RAC1) Activated Kinase 1; PD, Parkinson’s disease; PSD, post-synaptic density; HPtau, hyperphosphorylated Tau; Rac1, Ras-related C3 botulinum toxin substrate 1; TNFα, tumor necrosis factor α; TrkB, tyrosine receptor kinase B; VD, vascular dementia.

Figure 2 illustrates the plausible mechanistic pathways underpinning the alterations of hippocampal structural plasticity in neurodegenerative diseases. The majority of previous studies demonstrate that pathological molecules (Aβ plaques, Tau proteins, and α-Syn), mutated genes (*mhtt*), and neuroinflammation underpin the structural pathologies in hippocampal neurons. Therefore, targeted therapies against pathological molecules, mutated genes, and neuroinflammation, which restore hippocampal structural pathologies, may offer a novel mechanism to alleviate the symptoms of neurodegenerative diseases.

## 5. Hippocampal Dysfunction and Structural Plasticity in the Aging Brain

Apart from the neurodegenerative conditions, hippocampal dysfunction and cognitive impairment are often associated with aging [398,399,400,401]. Age-related hippocampal dysfunction is a broad topic and a major risk factor for neurodegenerative diseases, such as AD [402]. Several recent review articles offer excellent information on the involvement of hippocampal dysfunction in the context of aging [402,403,404,405,406].

Age-dependent aberrations of hippocampal dendritic complexity are well documented [407,408], with older age being directly correlated with impairments in dendritic complexity. Sex-related factors include female mice being less susceptible to aging-related dendritic complexity, at least in the CA1 region [409]. The influence of sex hormones and aging on hippocampal structural plasticity is adeptly discussed by Galea et al. [56].

Moreover, many studies described decreased dendritic spine density in aged mice hippocampi [407,408]. In contrast, another research group described unchanged spine density in the hippocampus over aging [409]. However, the influence of other factors like sex hormones cannot be excluded in these incongruent observations [56]. Detailed regional susceptibilities in aging-related dendritic spine pathology in the hippocampus have already been discussed previously [57]. Collectively, complex heterogeneous neurodegenerative mechanisms are presumably involved in alterations of hippocampal structural plasticity in aging brains.

## 6. Concluding Remarks

The hippocampus is one of the brain areas that shows neuronal remodeling in many neurodegenerative diseases, including AD, PD, HD, and MS. Neuronal remodeling proceeds through structural changes in the dendrites and spines of hippocampal neurons. The current review presented an overview of structural modifications of the hippocampus in neurodegenerative diseases, along with the underlying mechanisms. This review confirmed that hippocampal neuroarchitecture is affected at different levels (functional and structural) and to varying degrees in these neurodegenerative diseases.

In AD, alterations in dendritic complexity and spine density of hippocampal neurons start appearing in the early phase of disease progression. Moreover, the main molecular cues underlying these morphological changes in hippocampal neurons are Aβ plaques and HPtau, which alter the microtubule arrangement in the cells. The majority of the studies regarding PD elaborate on structural impairment in the context of striatal MSNs. Nevertheless, a few recent studies have shifted the focus to the hippocampus. In HD, dendritic spine loss in CA1 is the main neuroplasticity change in the hippocampus. Up-to-date evidence suggests that *mhtt* is responsible for these observed structural changes in HD, which is caused by impaired synaptic energy metabolism caused by decreased mitochondrial function. As the dendritic growth is highly sensitive to the supply of ATP, the impaired energy metabolism in HD may influence neuronal dendritic arbor formation. In MS, alterations in structural plasticity led to severe demyelination and synaptic abnormalities in the hippocampus. Activated inflammatory cells and impaired brain activity could be the main factors responsible for the changes in dendritic arborization and spine density in the hippocampi of patients with MS and animal models. In VD, inflammation and the activation of kinases upon chronic hypoperfusion are associated with structural changes in both dendritic arbors and spines. However, available literature on FTD and ALS is insufficient to make judgments on structural plasticity changes in these diseases. Collectively, different aspects of hippocampal structural plasticity were greatly affected in neurodegenerative diseases that are discussed in the current review. The negative changes in structural plasticity in the hippocampus are positively correlated with disease severity and further impact the functional symptoms in neurodegenerative diseases. However, there are still many gaps in understanding how the functional and structural changes of hippocampal neurons occur in these neurodegenerative diseases.

Consequently, this review provided an update on the status of the understanding of the role of hippocampal structural plasticity in the etiopathogenesis of neurodegenerative diseases. The current focus and existing gaps in the knowledge of hippocampal neuroplasticity in neurodegenerative diseases are also identified in this review. Conclusively, the information presented in this review may serve as a starting point for future mechanistic and therapeutic research in the field.

## Figures and Tables

**Table 1:** Recent evidences of the alteration of structural plasticity in the hippocampus in neurodegenerative diseases.

	Model	Dendritic Complexity	Spine Density/ Morphology	Mechanism	Ref.
AD	Clinical patients	Decreased	Decreased		[279]
	APP/PS1-TG mice	Decreased	Decreased spine density and mushroom		[114]
	APP/PS1-TG mice	Decreased		Fibrillary amyloid deposition	[277]
	APP/PS1-TG mice	Decreased		Fibrillary amyloid deposition	[277]
	APP/PS1-TG mice	Decreased	Decreased	Aβ plaques pathology	[280]
	APOE4-TG mice	Decreased	Decreased		[281]
	miR-34c-transfected mouse primary cultured hippocampal neurons	Decreased	Decreased density and filopodia		[282]
	N-tau-TG mice	Increased	Increased		[283]
	TgCRND8 mice	No change	Decreased, thin, and stubby	Aβ plaques pathology Alteration of GluR	[112]
	2576-TG mice		Decreased	Aβ plaques pathology Reactive gliosis	[116]
	Aβ-infused rats		Decreased		[113]
	Aβ-infused rats		Decreased spine density and length Decreased, thin, and filopodia	Glu circulation	[115]
	Aβ-treated rat hippocampal slices		Decreased	NMDAR	[117]
	APP/PS1-TG mice		Decreased spine length Increased neck size		[284]
	APP-knock-in mice		Decreased mushroom	Downregulation of synaptic STIM2–nSOC–CaMKII pathway	[285]
PD	LRRK2-mutant mouse primary cultured hippocampal neurons	Decreased		LRRK2 regulation	[286]
	LRRK2-mutant mice	Decreased		LRRK2 regulation	[287]
	LRRK2-mutant mice	Decreased	Decreased	LRRK2 regulation	[288]
	MPTP-lesioned mice		Decreased		[139]
	MPTP-lesioned mice	No change	Decreased		[289]
	PFF-treated primary cultured hippocampal neurons		Decreased mushroom	α-synucleinopathy	[290]
	PFF-treated primary cultured hippocampal neurons		Decreased spine density and mushroom	α-Syn induced dysregulation of the actin-binding protein	[291]
HD	R6/1-TG mice	Decreased			[292]
	Hip14-deficient mice		Decreased		[293]
	R6/1-TG mice		Decreased	NF-κB signaling	[294]
	Pyk2-deficient mice		Decreased	Pyk2 regulation	[172]
	R6/1-TG mice		No change		[295]
MS	EAE mice	Decreased			[296]
	EAE mice	Decreased			[297]
	Cuprizone-diet fed mice		No change in total density Increased mushroom		[298]
VD	BCCAO rats	Decreased	Decreased spine density and mushroom		[65]
	BCCAO rats	Decreased	Decreased	APP	[299]
	BCCAO rats		Decreased		[300]
	BCCAO rats		Decreased spine density and mushroom		[301]
	BCCAO rats		Decreased	Suppression of AMPK pathway	[302]
ALS/FTD	TDP-43 overexpressing primary cultured hippocampal neurons	Decreased		RNA-binding function of TDP-43	[67]
ALS	SOD1G93A TG mice	No change	No change		[303]

Abbreviations: AD, Alzheimer’s disease; ALS, amyotrophic lateral sclerosis; Aβ, amyloid β; APP/PS1, amyloid precursor protein/presenilin 1; AMPK, AMP-activated protein kinase; APOE4, apolipoprotein E4; α-Syn, alpha-synuclein; BCCAO, bilateral common carotid artery occlusion; BDNF, brain-derived neurotrophic factor; CaMKII, calmodulin-dependent protein kinase II; EAE, experimental autoimmune encephalomyelitis; FTD, frontotemporal dementia; Glu, glutamate; GluR, glutamate receptor; HD, Huntington’s disease; LRRK2, leucine-rich repeat kinase 2; miR, microRNA; MPTP, 1-methyl-4-phenyl-1,2,3,6-tetrahydropyridine; MS, multiple sclerosis; NF-κB, nuclear factor kappa B; NMDAR, N-methyl D-aspartate receptor; PD, Parkinson’s disease; PFF- α-Syn preformed fibrils; Pyk2, proline-rich tyrosine kinase 2; STIM2, stromal interaction molecule 2; TDP-43, TAR DNA binding protein-43; TG, transgenic; TgCRND8, transgenic CRND8; VD, vascular dementia.

## Data Availability

This paper utilized original data not used in other publications. The datasets generated and/or analyzed in the present study are available from the corresponding author upon reasonable request.
